# Fitness and Adiposity Are Independently Associated with Cardiometabolic Risk in Youth

**DOI:** 10.1155/2013/261698

**Published:** 2013-07-31

**Authors:** Duncan S. Buchan, John D. Young, Lynne M. Boddy, Robert M. Malina, Julien S. Baker

**Affiliations:** ^1^Institute for Clinical Exercise and Health Science, School of Science, University of the West of Scotland, Hamilton ML3 0JB, UK; ^2^The Physical Activity Exchange, Research Institute for Sport and Exercise Sciences, Liverpool John Moores University, 62 Great Crosshall Street, Liverpool L3 2AT, UK; ^3^Department of Kinesiology and Health Education, University of Texas at Austin, Austin, TX D3700, USA; ^4^Department of Kinesiology, Tarleton State University, Stephenville, TX 76402, USA

## Abstract

*Purpose*. The purpose of the study was to examine the independent associations of adiposity and cardiorespiratory fitness with clustered cardiometabolic risk. *Methods*. A cross-sectional sample of 192 adolescents (118 boys), aged 14–16 years, was recruited from a South Lanarkshire school in the West of Scotland. Anthropometry and blood pressure were measured, and blood samples were taken. The 20 m multistage fitness test was the indicator of cardiorespiratory fitness (CRF). A clustered cardiometabolic risk score was constructed from HDL-C (inverted), LDL-C, HOMA, systolic blood pressure, and triglycerides. Interleukin-6, C-reactive protein, and adiponectin were also measured and examined relative to the clustered cardiometabolic risk score, CRF, and adiposity. *Results*. Although significant, partial correlations between BMI and waist circumference (WC) and both CRF and adiponectin were negative and weak to moderate, while correlations between the BMI and WC and CRP were positive but weak to moderate. Weak to moderate negative associations were also evident for adiponectin with CRP, IL-6, and clustered cardiometabolic risk. WC was positively associated while CRF was negatively associated with clustered cardiometabolic risk. With the additional adjustment for either WC or CRF, the independent associations with cardiometabolic risk persisted. *Conclusion*. WC and CRF are independently associated with clustered cardiometabolic risk in Scottish adolescents.

## 1. Introduction

Although prediction of cardiovascular disease (CVD) risk was previously considered pertinent to adults, it is now accepted as a paediatric problem since the pathological processes begin in childhood [1, 2]. Clustering of cardiometabolic risk factors such as high blood pressure, insulin resistance, elevated triglycerides, central obesity, low cardiorespiratory fitness (CRF), physical inactivity, and lowered HDL is routinely noted among both children and adolescents [1–3]. Of relevance to potential effects of long term exposure to the clustering of risk factors to adulthood morbidity and/or mortality, recent evidence indicates that a number of risk factors associated with CVD in later life track from adolescence and predict the extent of atherosclerotic disease in adulthood [4, 5]. Measurement of risk in youth is thus potentially important as early detection may help to identify individuals who may be susceptible to developing CVD-related disorders.

Obesity is a major contributor to poor risk profiles among youth [6, 7]. Whilst obesity and overweight are associated with increased cardiometabolic risk in adolescents [8], moderate to high levels of CRF are also associated with a reduction in clustering of cardiometabolic risk factors [1, 3]. Although poor CRF and excess adiposity often occur concurrently [8], the relative importance of each to the prediction of cardiometabolic risk requires further study. The potential independent effect of adiposity and CRF on cardiometabolic risk is receiving increased attention [9, 10]. However, other more recently identified risk factors for CVD may also be relevant for the improvement of risk prediction. Adipocytokines such as interleukin-6 (IL-6) and adiponectin as well as inflammatory cytokines such as C-reactive protein (CRP) are associated with the development of atherosclerosis and type 2 diabetes in adults [11]. Since obesity is considered an independent risk factor for CVD, it is not surprising that both adipocytokines have been associated with poor cardiometabolic risk profiles in youth [12, 13]. Similar findings are also evident for CRP [14].

Given the associations among risk factors in adults, evidence dealing with the clustering of established and more recently identified risk factors among youth is limited. Thus the purpose of this study was twofold: first, to investigate the joint associations between measures of adiposity, CRF, IL-6, CRP, and adiponectin and clustered cardiometabolic risk among youth and, second, to examine the independent associations between adiposity and CRF with clustered cardiometabolic risk. Risk factors often track from adolescence into adulthood [5, 15], so information on their relationships is potentially relevant to the development and implementation of preventive measures and effective intervention strategies.

## 2. Methods

### 2.1. Sample

A cross-sectional sample of volunteers (118 boys, 74 girls, 15.0 ± 2.6 years) was recruited from South Lanarkshire schools in the West of Scotland. Informed consent was obtained from all students and their parents. The study was approved by the ethics committee of the University of the West of Scotland. All tests were undertaken between 9:00 a.m. and noon while all measurements were taken by the same individuals.

### 2.2. Physical and Physiological Measures

Barefoot stature was measured to the nearest 1 mm (Seca Stadiometer, Seca Ltd., Birmingham, UK). Weight in light indoor clothing, without shoes, was measured to the nearest 0.1 kg using calibrated electronic weighing scales (Seca 880, Digital Scales, Seca Ltd., Birmingham, UK). Body mass index (BMI) was calculated (weight/height^2^, kg/m^2^). Waist circumference (WC), an index of central (abdominal) adiposity, was measured at the level midway between the lower ribs and iliac crest [16]. Sexual maturation status was self-assessed using the criteria for pubic hair [17]. 

Systolic and diastolic blood pressures (SBP and DBP) were measured with an automated monitor (Omron M10-IT Blood Pressure Monitor HEM-7080IT-E, Omron Healthcare UK Ltd., Milton Keynes, UK) after each participant sat quietly for 10 min. The average of the second and third measures was retained for analysis.

CRF was measured using the 20 m multistage fitness test (20MSFT, number of completed shuttles) which is viewed as a valid predictor of maximal CRF in young people [18]. Specific details of the 20MSFT protocol have been described elsewhere [19]. Participants were instructed to run until exhaustion and were given verbal encouragement throughout the test.

### 2.3. Blood Sampling

Blood samples were taken between 9:00 and 11:00 a.m. after an overnight fast. Fasting was verified prior to sampling. Qualified phlebotomists, experienced in paediatric sampling techniques, obtained all blood samples. Blood samples were obtained from an antecubital vein and collected in a BD Vacutainer plasma tube (Becton, Dickinson and Company, Franklin Lakes, USA). Plasma was isolated by centrifugation at 3,500 rpm for 10 minutes and frozen at −80°C within two hours of collection. Analyses were completed within three months of collection. Insulin, HDL-C, LDL-C, CRP, glucose, IL-6, adiponectin, the homeostasis model assessment (HOMA), and triglycerides (TG) were measured with standard procedures. Triglycerides were measured by enzymatic methods (TR210 and CH200 Randox, Co. Antrim, UK) and a Camspec M107 spectrophotometer (Camspec, Leeds, UK). Concentration of HDL-C was determined after precipitation of very-low-density and low-density lipoproteins by the addition of phosphotungstic acid in the presence of magnesium ions. The Friedewald formula [20] was used to calculate LDL-C concentration. Glucose was measured using the glucose oxidase method (GL364, Randox, Co. Antrim, UK) and analyzed with a Camspec M107 spectrophotometer (Camspec, Leeds, UK). Plasma insulin was analyzed with commercially available immunoassay kits (ALPCO, Salem, NH, USA) and a Camspec M107 spectrophotometer (Camspec, Leeds, UK). Concentrations of IL-6 and CRP were measured with specific enzyme linked immunosorbent assay (ELISA) kits (R & D Systems, Abingdon, UK) and an MRX microplate reader (Dynatech Laboratories, Cambridge, MA, USA). HOMA calculation was used as an indication of insulin resistance which was calculated as the product of fasting glucose (mmol/L) and insulin (*μ*U/mL) divided by the constant 22.5 [21]. 

### 2.4. Clustered Cardiometabolic Risk Score

Since differences within individual risk markers among youth may be difficult to identify [1], a clustered risk score for each participant was constructed from HDL-C (inverted), LDL-C, HOMA, SBP, and TG. These variables were selected as all are well-established risk factors [1, 2]. A *z*-score (*z* = (value − mean)/SD) for each variable was constructed separately for boys and girls and by 1 yr age groups. The *z*-scores of the individual risk factors were then summed to create a clustered cardiometabolic risk score for each participant with a lower score indicating a healthier overall risk profile.

### 2.5. Data Analysis

All analyses were undertaken using Statistical Package for the Social Sciences (version 18; SPSS Inc., Chicago, IL, USA), with values of *P* < 0.05 considered statistically significant. Data was checked for normality of distribution before the analysis and transformed where appropriately. Consequently, BMI, insulin, HOMA, adiponectin, CRF, HDL, LDL, SBP, triglycerides, and IL-6 were log-transformed before *z*-scores (adjusted for sex and 1 yr age groups) were constructed. Untransformed data are presented throughout for ease of interpretation. Complete data for all variables was available for 64% of the sample. Multiple imputations were used to address the potential bias from missing data. Missing data rarely occur completely at random, and omitting participants with one or more missing values may lead to bias and reduction in statistical power. Consequently, missing values were replaced through multiple imputations. Five sets of imputations using all variables in the model were created; the imputation was then aggregated across the 5 datasets; the pooled data were subsequently used. Multiple imputation is recommended for epidemiological studies with extensive missing values, as long as these amount to <60% of the total [22]. In the present study, the extent of “missingness” was well within this limit (<10%).

Descriptive data are presented as means and standard deviations. Significant differences between the sexes were determined using ANOVA, whereas differences in maturity status between the sexes were analysed using the chi-squared test. As no significant interaction was found for age, maturation, and sex, all analyses were performed with boys and girls together to increase statistical power. For variables where the assumptions of normality and/or homogeneity of variance were invalid, the nonparametric Mann-Whitney *U* test was used. Partial correlations were used for bivariate associations between adiponectin, IL-6, CRP, WC, BMI, and CRF and clustered cardiometabolic risk. Multiple regression analysis was used to examine the relationships between CRF, WC and clustered cardiometabolic risk through two separate models. Model 1 included WC or CRF and was adjusted for sex, age, and maturity status. Model 2 included either WC or CRF and was additionally adjusted for independent associations of the two predictor variables.

## 3. Results

Participant characteristics are summarized in Table 1. Boys were taller and heavier than girls (*P* < 0.001), while BMI was similar between genders. Boys had a larger WC (*P* < 0.05) and higher CRF (*P* < 0.001) than girls. Girls had higher levels of HDL-C (*P* < 0.001) but lower levels of LDL-C (*P* < 0.05) than boys. Partial correlations for measures of adiposity, CRF, metabolic markers of CVD risk, and clustered cardiometabolic risk are shown in Table 2. Weak to moderate negative associations were evident for BMI and WC with both CRF and adiponectin and for adiponectin with CRP, IL-6, and clustered cardiometabolic risk score. Weak to moderate positive associations were observed between BMI and WC and CRP.

Results of the multiple regression analyses are summarized in Table 3. WC was positively associated with clustered cardiometabolic risk (*P* < 0.001), while CRF was negatively associated with clustered cardiometabolic risk (*P* < 0.001). With the additional adjustment in model 2 for either WC or CRF, the observed associations remained significant (*P* < 0.001).

## 4. Discussion

Independent relationships among two indicators of adiposity, CRF, metabolic risk factors, and clustered cardiometabolic risk were initially evaluated. BMI and WC were negatively associated with both CRF and adiponectin, although only BMI was positively associated with CRP. Adiponectin was negatively associated with CRP, IL-6, and the clustered cardiometabolic risk score. Although the associations were significant, they were weak and at best moderate. This may suggest that sufficient time may not have elapsed for the relationships to fully develop. 

Multiple regression models were then used to evaluate the independent associations of WC and CRF with clustered cardiometabolic risk. Both were significantly related to clustered cardiometabolic risk in this sample of Scottish adolescents (Table 3).

Adiponectin was inversely related to both WC and BMI in Scottish adolescents, consistent with other studies [13, 23]. This is not surprising since adiponectin is an atypical adipokine with lower levels in obese and overweight individuals [24]. The inverse association among adiponectin, CRP, and IL-6 was also consistent with the literature [25, 26]. Adiponectin is known to play a protective role in the development of insulin resistance and diabetes, both of which are positively associated with elevated levels of CRP [27] and IL-6 [28]. Moreover, adiponectin is an anti-inflammatory adipokine known to indirectly decrease levels of CRP and IL-6 through the dose-dependent, reciprocal inhibition of tumour necrosis factor-*α* (TNF-*α*) thus inhibiting these proinflammatory cytokines [26]. Consequently, observations in the present study may indicate that decreased production of adiponectin contributes to the systemic and vascular inflammation commonly found in obesity through its involvement within the regulatory pathway of several factors including TNF-*α*, IL-6, CRP, insulin resistance, body composition, and disease states.

Interestingly, only adiponectin was associated (negatively) with the clustered cardiometabolic risk score in this apparently healthy sample. Previous investigations in youth have demonstrated positive associations between CRP, and not IL-6, and clustered cardiometabolic risk [29]. A plausible explanation for the lack of association between the cardiometabolic risk score and both CRP and IL-6 in the present study may lie in the apparent health of the cohort. As IL-6 is produced and secreted from adipose tissue, which in turn stimulates CRP production from the liver, one would expect elevated levels of both indicators in the presence of obesity, increasing the likelihood of significant associations with clustered cardiometabolic risk. Mean baseline IL-6 data in the sample (Table 1) was nearly 4 times greater than that in the cohort used by Andersen and colleagues (2010). The sample of Scottish adolescents was, on average, 5 years older than the sample described by Andersen and colleagues [29]. It is possible that age per se and advanced sexual maturity status contributed to the variable results. Nevertheless, the clustering of cardiometabolic risk factors may have multiple causes independent of inflammation. Indeed, the appearance of cardiometabolic risk may occur before high levels of inflammatory markers are evident. 

The associations between adiponectin and clustered cardiometabolic risk have also been previously noted in youth [30, 31] although the present study may be the first, to the best of our knowledge, to document similar associations in Scottish youth. This is important given ethnic and gender differences in adiponectin levels among cohorts [23, 32]. Further understanding of factors associated with clustered risk scores is important, but hypoadiponectinemia and low-grade inflammation may act upon clustered cardiometabolic risk differently and independently.

Results of the regression analyses (Table 3) indicated significant associations between CRF and WC and clustered cardiometabolic risk independently of sex, age, and sexual maturity status. The findings were consistent with other studies showing an association between higher levels of CRF and a healthier risk profile in children and adolescents [1, 29, 33–35]. Previous investigations have also demonstrated a negative relationship between CRF and measures of adiposity [36] and a stronger relationship between measures of adiposity and CVD risk than that between CRF and clustered risk [37, 38]. Although CRF was associated with measures of adiposity in this sample (Table 2), results of the multiple regression analyses (Table 3) indicated that neither CRF nor WC was more strongly associated with clustered cardiometabolic risk.

Significant associations between WC (positive) and CRF (negative) and clustered cardiometabolic risk were noted, but the strength of the associations was enhanced, albeit marginally, after controlling for either CRF or WC in the second regression model. The finding that WC was independently associated with clustered cardiometabolic risk, after controlling for CRF, was consistent with some [3, 37] but not all [36, 39] studies of youth. Potential explanations for the variable results may relate to sampling variation, measurement protocols, and indicators of risk used in the different studies. Although the observations of this study are limited due to its cross-sectional nature, the strength of the present study is the measurement of traditional CVD risk factors in addition to adiponectin, IL-6, and CRP in a young cohort with findings indicating that measures of fatness may have different associations with markers of inflammation and that both WC and CRF act independently upon components of the cardiometabolic risk score. 

## 5. Conclusion

Few studies have examined the relationships between adiponectin, inflammatory cytokines, and clustered cardiometabolic risk in adolescents. The findings indicated an association between adiponectin and clustering of CVD risk factors. Given the pleiotropic effects of adiponectin, further research is needed to establish the utility of adiponectin as an independent indicator of cardiometabolic risk within diverse youth populations. The relative importance of adiposity and CRF in the management of cardiometabolic risk was also noted in this sample of Scottish youth.

## Figures and Tables

**Table 1:** Participant characteristics (means ± standard deviations, except for stage of sexual maturation).

	Boys (*n* = 118)	Girls (*n* = 74)	*P* value
Age (yr)	15.14 ± 2.57	14.62 ± 2.78	0.186
Stage of pubic hair (*n*) I/II/III/IV/V	8/20/8/38/44	14/18/26/12/4	<0.001
Height (cm)	166.58 ± 20.96	150.85 ± 37.65	<0.001
Weight (kg)	63.22 ± 17.46	54.87 ± 15.52	<0.001
BMI (kg m^−2^)	22.49 ± 4.98	23.58 ± 7.78	0.455
WC (cm)	71.26 ± 9.89	67.69 ± 9.99	0.016
SBP (mmHg)	118.07 ± 23.59	117.78 ± 10.69	0.106
DBP (mmHg)	68.88 ± 15.75	68.38 ± 9.94	0.464
CRF (shuttles)	78.89 ± 25.40	41.72 ± 15.73	<0.001
HDL-C (mMol L^−1^)	1.66 ± 0.70	2.02 ± 0.78	<0.001
LDL-C (mMol L^−1^)	1.71 ± 1.51	1.14 ± 1.44	0.013
TG (mMol L^−1^)	0.81 ± 0.37	0.86 ± 0.39	0.395
IL-6 (pg mL^−1^)	4.44 ± 8.00	6.91 ± 12.54	0.304
Adiponectin (mg L^−1^)	9.87 ± 5.93	10.19 ± 6.96	0.222
Glucose (mMol L^−1^)	4.49 ± 1.03	4.37 ± 1.10	0.456
CRP (mg L^−1^)	1.71 ± 2.25	1.95 ± 2.19	0.479
Insulin (IU mL^−1^)	6.54 ± 5.60	7.94 ± 5.96	0.052
HOMA	1.33 ± 1.45	1.53 ± 1.14	0.105

BMI: body mass index; WC: waist circumference; SBP: systolic blood pressure; DBP: diastolic blood pressure; CRF: cardiorespiratory fitness; HDL-C: high-density lipoprotein cholesterol; LDL-C: low-density lipoprotein cholesterol; TG: triglycerides; IL-6: interleukin-6; CRP: C-reactive protein; HOMA: homeostasis model assessment.

**Table 2:** Partial correlations (controlling for age, sex, and sexual maturity status) between the clustered cardiometabolic risk score (SBP + HDL-C + LDL-C + HOMA + TG), BMI, WC, CRF, adiponectin, CRP, and IL-6.

	BMI	WC	CRF	Adiponectin	CRP	IL-6
BMI	—					
WC	0.47*	—				
CRF	−0.27*	−0.29*	—			
Adiponectin	−0.28*	−0.20**	0.08	—		
CRP	0.19**	0.12	0.01	−0.21***	—	
IL-6	0.04	0.05	−0.11	−0.30*	0.14	—
MRS	0.04	0.01	0.02	−0.22***	0.28	0.13

BMI: body mass index; WC: waist circumference; CRF: cardiorespiratory fitness; IL-6: interleukin-6; CRP: C-reactive protein; MRS: clustered cardiometabolic risk score. *P < 0.001, **P < 0.05, ***P < 0.01.

**Table 3:** Standardized regression coefficients examining the association of waist circumference and cardiorespiratory fitness with clustered cardiometabolic risk.

	Waist circumference				Cardiorespiratory fitness			
	*R* ^2^	*β*	*R* ^2^ change	*P* value	*R* ^2^	*β*	*R* ^2^ change	*P* value
Model 1	0.570	0.002	0.324	<0.001	0.571	−0.073	0.326	<0.001
Model 2	0.571	0.017	0.002	<0.001	0.571	−0.078	0.000	<0.001

Model 1 is adjusted for sex, age, and sexual maturity status. Model 2 is adjusted for all covariates and either waist circumference or cardiorespiratory fitness.
